# Occupational Class Differences in Emotional Exhaustion Among Municipal Employees – The Role of Employment Sector and Psychosocial Working Conditions

**DOI:** 10.1177/00332941221106393

**Published:** 2022-06-01

**Authors:** Jouni Lahti, Jade Knop, Tea Lallukka, Jaakko Harkko, Anne Kouvonen

**Affiliations:** Faculty of Social Sciences, 154260 University of Helsinki, Finland; Department of Public Health, University of Helsinki, Finland; Department of Public Health, University of Helsinki, Finland; Faculty of Social Sciences, 154260 University of Helsinki, Finland; Faculty of Social Sciences, 154260 University of Helsinki, Finland; Centre for Public Health, Queen’s University Belfast, UK

**Keywords:** burnout, stress, socioeconomic position, working conditions, employment sector

## Abstract

Studies examining occupational class differences in burnout symptoms across employment sectors are scarce. The aim of this study was to examine whether occupational class is associated with emotional exhaustion, and whether there are differences in the examined associations between employment sectors. A further aim was to examine to which extent psychosocial working conditions may explain these associations. Survey data were collected in 2017 among 19–39-year-old employees of the City of Helsinki (4630 women and 1267 men, response rate 51.5%). Occupational class included four classes: 1. manuals, 2. routine non-manuals, 3. semi-professionals, 4. managers and professionals. Employment sector was classified into three groups: 1. health and social care, 2. education and 3. ‘other’. Linear regression analysis and IBM SPSS 25 statistical program were used. The analytical sample included 4883 participants. The highest occupational class, i.e. managers and professionals, reported the highest emotional exhaustion. In terms of the sector, those working in education had the highest scores of emotional exhaustion. The associations between occupational class and emotional exhaustion differed somewhat between the sectors. Adjustment for job demands attenuated the differences in emotional exhaustion between occupational classes, whereas adjustment for job control and job strain widened the differences. Attention should be paid to occupations with excess mental demands, and to employees in the education sector, who showed the highest risk of emotional exhaustion.

## Introduction

Burnout is an increasing health problem having negative effects on individuals, organisations and the society (Salvagioni et al., 2017; Ahola et al., 2006). Burnout is a disorder caused by prolonged work stress gradually exhausting the resources of an individual. Poor recovery appears to mediate the stress-burnout relationship (Gluschkoff et al., 2016). Burnout can lead to various psychological and physical symptoms, such as tiredness, concentration difficulties, sleep problems, depressive symptoms as well as somatic symptoms such as headache and back pain (Schaufeli & Enzmann, 1998). Besides affecting individual life satisfaction and quality of life (Hakanen et al., 2012), burnout increases sickness absences (Ahola et al., 2008) and decreases work productivity (Demerouti et al., 2009), causing major costs at organisational and societal levels.

### Burnout and Emotional Exhaustion

According to the three-dimensional definition of burnout, it is a syndrome characterized by emotional exhaustion, a cynical attitude toward work, and a decline in professional efficacy (Maslach & Jackson, 1981), including an assumption about how these different dimensions evolve over time. Typically, it is thought that emotional exhaustion is the first sign of burnout. Feelings of cynicism develop as a means for the employee to cope with an exhausting situation. As a result of emotional exhaustion and cynicism, the employee’s experience of his or her professional skills can deteriorate. This sequence has received the most empirical support, although different sequences have also been proposed (Toppinen-Tanner et al., 2002; Schaufeli et al., 2020). For instance, as cynical attitude toward work is a coping mechanism and decline of professional efficacy is a cause of stress, it has been suggested that these dimensions should be examined separately (Kristensen et al., 2005). In this study, we focus on emotional exhaustion as it is the core dimension of burnout (Kristensen et al., 2005; Seidler et al., 2014; Schaufeli et al., 2020).

### Occupational Class and Work Environment in Relation To Burnout

Factors related to working environment have been widely investigated in relation to burnout and according to a systematic review and meta-analyses (Aronsson et al., 2017) and a review (Seidler et al., 2014), adverse psychosocial working conditions, such as excessive job demands and especially low job control, increase the risk of burnout and emotional . A recent longitudinal study examined bidirectional associations, suggesting that adverse psychosocial working conditions are leading to burnout rather than the opposite direction (Shahidi et al., 2022). The significance of structural factors, however, has received less attention. For instance, the association between occupational class and burnout has been rarely examined showing generally relatively weak and inconsistent findings (Ahola et al., 2006; Soares et al., 2007; Norlund et al., 2010). Furthermore, a study regarding diverse industry sectors in Switzerland showed (Hämmig & Bauer, 2013) that lower occupational class was associated with poorer self-rated health and increased sickness absence while higher class was associated with increased symptoms of burnout and exhaustion. In addition, a previous study conducted among Finnish public sector employees showed that higher occupational class was associated with increased symptoms of exhaustion (Helkavaara et al., 2011).

Previous studies have concentrated on examining occupations and sectors believed to have a high risk of burnout, such as employees in health and social services and in education (Aronsson et al., 2017). Numerous studies have shown that differences in psychosocial working conditions contribute to socioeconomic differences in health (Hoven and Siegrist 2013; Rahkonen et al., 2006). However, we lack studies on occupational class differences in emotional exhaustion across and between employment sectors and the contribution of psychosocial working conditions. Adverse psychosocial working conditions, such as high job strain defined as simultaneous low job control and high job demands may predispose an employee to stress (Aronsson et al., 2017). Adverse psychosocial working conditions have been found to be unequally distributed between occupational groups and employment sectors (Hämmig & Bauer, 2013).

### The Aim of the Study

The aim of this study was to examine whether occupational class is associated with emotional exhaustion among municipal employees, and whether there are differences in the examined associations between employment sectors. A further aim was to examine to which extent psychosocial working conditions may explain the examined associations. According to previous knowledge, we hypothesized that higher occupational class is associated with increased emotional exhaustion, and that the employment sector modifies the association as the working conditions associated with occupational classes may vary across sectors. In addition, we expect that the associations between occupational class and emotional exhaustion are partly mediated by psychosocial working conditions.

## Materials and Methods

### Data

Survey data were collected in autumn 2017 among employees of the City of Helsinki who were aged 19 to 39 years (born in 1978 or later) and had at least a half-time (50% of working hours) employment contract and employment lasting for at least 4 months. The City of Helsinki is the largest employer in Finland with approximately 39,000 employees annually. The City of Helsinki provides basic services for its residents, such as education, health and social care, and public administration. Thus, the employees represent wide range of occupational titles from different employment sectors. Survey data were collected mainly via secure online server, and in addition, postal questionnaires were mailed to those without email addresses or who did not respond online. Furthermore, for non-respondents to the online and postal surveys, data on a selected range of variables were collected with telephone interviews. The final response rate was 51.5% and the number of participants was 5897 (4630 women and 1267 men). The present study only includes online and postal survey respondents (n = 5111), as all the variables of interest such as emotional exhaustion were not included in the telephone interview. Due to missing information on study variables (n = 227), the final analytical sample included 4884 participants. The gender distribution matches that of the target population, and in general that of the municipal sector in Finland. The non-response analysis showed that the data are broadly representative of the target population with respect to key variables, i.e., sociodemographic and work-related factors and health (Lallukka et al., 2020). The ethics committee of the Faculty of Medicine, University of Helsinki gave their approval for the study protocol. Additionally, the City of Helsinki provided permission to conduct this study.

### Emotional Exhaustion

Emotional exhaustion was measured by a subscale inventory developed from the Maslach Burnout Inventory (MBI) (Maslach & Jackson, 1981) at the Finnish Institute of Occupational Health (Kalimo and Tappinen, 1997). A question concerning job frustration and two questions concerning working with clients and patients were omitted from the original nine questions, as they were specific to human service work and our sample consisted of a wide range of occupations. Consequently, the inventory included the following six items: 1. I feel totally worn out after a day at work, 2. I feel tired in the morning when I have to get up and go to work, 3. I have to work too hard, 4. I feel like I’m totally exhausted, 5. My work is definitely too stressful, and 6. I worry about my work even when I am off duty. Response alternatives ranged on a five-point Likert scale from very seldom to very often. Mean scores were calculated and the final exhaustion mean score ranged from 1 to 5. Those responding to five or all six items were included. Cronbach’s alpha for the internal consistency was high (0.89).

### Occupational Class

Occupational class was categorized using occupational information derived from the personnel register of the City of Helsinki for those participants who gave a written consent for such data linkage (83%) and for the rest, information on occupations was obtained from the survey. Four classes were included: 1. Managers and professionals, including those with subordinates and those doing managerial/administrative work as well as other upper non-manual employees doing professional work, such as teachers and doctors; 2. Semi-professionals, including nurses, foremen, technicians, and other intermediate non-manual employees; 3. Routine non-manual employees, including non-professional employees doing, for example, clerical work as well as other lower non-manual employees, e.g. nursery workers and care workers; and 4. Manual workers, including those in transport work and other technical services as well as those employed in cleaning work in the health and social care sector (Lallukka et al., 2020).

### Psychosocial working conditions

Job demands and job control were measured by Karasek’s job strain questionnaire (Karasek et al., 1998). The demand and control scales were separately summed up and weighted according to Karasek’s instructions. The weighted mean scores were calculated for those responding to over 50% of the items, i.e. at least three items for job demands and five items for job control. Cronbach Alpha for job demands was 0.72 and for job control 0.79. Job strain was formed by dividing the mean score of job demands with the mean score of job control providing a score 0.2–5. Each measure was classified into three groups according to the lowest and highest quartile cut-points.

### Employment Sector

Information on employment sector was derived from the personnel register of the City of Helsinki for those who gave permission to register linkage (83%) and for the rest, when applicable, the employment sector was completed from the questionnaire information on occupational title (missing n = 177). Three major employment sectors were formed: 1. Health and social care, including e.g. occupations in primary care, hospital services and prevention, social welfare services as well as elderly care; 2. Education, including e.g. occupations in primary and secondary school education as well as vocational training; 3. ‘Other’, including occupations in other sectors e.g. culture and leisure sector, transportation, rescue service, financial administration.

### Covariates

Age and gender were derived from the questionnaire and used as confounders.

### Statistical Analyses

The main analyses were conducted using linear regression analyses. Marginal mean scores and 95% confidence intervals of emotional exhaustion were estimated by occupational class to test the hypotheses. Statistically significant (*p* < 0.05) differences between occupational classes are indicated using the managers and professionals as reference group. An interaction test was conducted to test whether the association between occupational class and emotional exhaustion differed between employment sectors. The interaction between occupational class and employment sector was statistically significant (*p* < 0.001 for interaction). Analyses were performed for the whole sample and stratified by employment sector. In model 1, age and gender were adjusted for. In model 2, age, gender, and job demands; in model 3, age, gender, and job control; and in model 4, age, gender, and job strain were controlled for. IBM SPSS Statistics 25 was used for the analyses.

## Results

### Descriptive analyses

Age showed a weak association with emotional exhaustion, although somewhat lower scores in older participants (Table 1). Women (2.88) had a higher mean score of emotional exhaustion than men (2.52). Manual workers (2.58) had the lowest mean score and managers and professionals the highest (2.90). The education sector (2.97) had the highest mean score and the ‘other’ sector (2.56) the lowest. Of the psychosocial working conditions, job control showed the weakest association, while job demands and job strain showed a clear association with emotional exhaustion.

**Table 1.** Distribution of the study variables and their association with emotional exhaustion: mean scores with 95% confidence intervals.

	N	%	Emotional Exhaustion
All	4883		2.80, 2.78–2.83
Age			
19–29 years	1557	31.9	2.85, 2.80–2.90
30–34 years	1647	33.7	2.81, 2.76–2.86
35–39 years	1679	34.4	2.75, 2.71–2.80
Gender			
Women	3903	79.9	2.88, 2.85–2.91
Men	980	20.1	2.51, 2.45–2.57
Occupational class			
Managers and professionals	1334	27.3	2.90, 2.85–2.95
Semi-professionals	1928	39.5	2.77, 2.72–2.81
Routine non-manuals	1358	27.8	2.81, 2.76–2.86
Manual workers	263	5.4	2.58, 2.47–2.70
Employment sector			
Health and social care	2223	45.5	2.79, 2.75–2.83
Education	1617	33.1	2.97, 2.93–3.02
Other	1043	21.4	2.56, 2.50–2.62
Job demands			
High	1022	20.9	3.65, 3.60–3.70
Intermediate	2651	54.3	2.77, 2.74–2.80
Low	1210	24.8	2.16, 2.11–2.20
Job control			
Low	1348	27.6	2.97, 2.92–3.02
Intermediate	2394	49.0	2.77, 2.73–2.81
High	1141	23.4	2.69, 2.63–2.74
Job strain			
High	1223	25.0	3.43, 3.38–3.48
Intermediate	2441	50.0	2.81, 2.77–2.83
Low	1219	25.0	2.17, 2.13–2.22

The associations between covariates and occupational class are presented in Table 2. Following the typical gender distribution in municipal work, the vast majority of managers and professionals, semi-professionals and routine non-manuals were women whereas gender distribution was more even in manual occupations. Managers and professionals were older than employees representing the other occupational classes. The vast majority (93%) of manual workers worked in the ‘other’ sector, and none of them worked in education. Of the routine non-manuals, every fifth worked in the ‘other’ sector, while education and health and social care comprised about 40% of the participants, respectively. Of the semi-professionals, two thirds worked in health and social care, every fifth in education and every tenth in the ‘other’ sector. Of the managers and professionals, half worked in education, more than every fourth in health and social care and every fifth in the ‘other’ sector.

**Table 2.** The distribution (%) of study variables by occupational class.

Occupational Class	Managers and Professionals	Semi-professionals	Routine Non-manuals	Manual Workers
**Total (N = 4883)**	1334	1928	1358	263
Age				
19–29 years	18.3	35.3	39.8	34.8
30–34 years	39.6	33.5	29.2	29.9
35–39 years	42.1	31.3	31.0	35.2
Gender				
Women	78.1	84.9	81.7	43.9
Men	21.9	15.1	18.3	56.1
Employment sector				
Health and social care	27.6	67.4	39.5	6.8
Education	50.9	21.1	39.2	0
Other	21.5	11.5	21.3	93.2
Job demands				
High	22.0	22.6	19.9	8.4
Intermediate	56.1	51.0	57.5	52.5
Low	21.9	26.3	22.6	39.2
Job control				
Low	7.9	27.4	41.6	56.7
Intermediate	44.2	54.4	49.0	34.6
High	47.9	18.2	9.4	8.7
Job strain				
High	10.3	26.7	35.1	36.1
Intermediate	57.6	47.3	48.4	39.5
Low	32.2	26.0	16.5	24.3

The proportion of employees with high job demands was lowest among manual workers (8.4%), whereas among other occupational classes the distribution was similar (Table 2). The proportion of those with low job control and high job strain increased with lower occupational class. Low job control varied from 7.9% among managers and professionals to 56.7% among manual workers. Likewise, high job strain varied from 10.6% among managers and professionals to 36.1% among manual workers being similar among the routine non-manuals 35.1%.

### Occupational class and emotional exhaustion among municipal employees

For testing the hypotheses that higher occupational class is associated with emotional exhaustion, we calculated mean scores of emotional exhaustion and compared all other occupational classes with the highest group i.e. managers and professionals Accordingly, the results showed that compared to managers and professionals the lower occupational classes had significantly lower mean scores of emotional exhaustion. Manual workers had the lowest levels of emotional exhaustion, and routine non-manuals and semi-professionals had levels somewhat lower than managers and professionals, when adjusting for age and gender. Adjustment for job demands attenuated the associations and the difference between managers and professionals and manual workers diminished. In contrast, adjustment for job control widened the differences: manual workers had the lowest levels of emotional exhaustion, and routine non-manuals and semi-professionals had lower levels than managers and professionals. Similarly, adjustment for job strain widened the differences: manual workers had the lowest, and routine non-manuals and semi-professionals had lower levels of emotional exhaustion than managers and professionals (Table 3).

**Table 3.** Emotional exhaustion mean scores with 95% confidence intervals by occupational class adjusted for age, gender and psychosocial working conditions.

Occupational Class	Model 1	Model 2	Model 3	Model 4
Managers and professionals	2.91, 2.86–2.97	2.88, 2.84–2.92	3.00, 2.95–3.06	3.05, 3.01–3.10
Semi-professionals	2.75, 2.70–2.79*	2.75, 2.72–2.79*	2.74, 2.70–2.78*	2.75, 2.71–2.79*
Routine non-manuals	2.79, 2.74–2.84*	2.79, 2.75–2.83*	2.74, 2.69–2.79*	2.68, 2.63–2.72*
Manual workers	2.71, 2.60–2.83*	2.84, 2.74–2.94	2.62, 2.50–2.73*	2.57, 2.47–2.67*

Model 1 Age and gender.

Model 2 Model 1+ job demands.

Model 3 Model 1+ job control.

Model 4 Model 1 + job strain.

*significantly (*p*<0.05) different from the manager and professionals.

### Occupational class and emotional exhaustion by employment sector

For testing the hypotheses that employment sector modifies the association between occupational class and emotional exhaustion we first conducted an interaction test. Accordingly, the interaction test showed statistically significantly (*p* < 0.001 for interaction) different associations between occupational class and emotional exhaustion by employment sector. Stratified analyses showed that the magnitude of the associations between occupational class and emotional exhaustion varied between the sectors (Table 4). In the health and social care sector, semi-professionals had a lower level of emotional exhaustion than the other occupational classes. Managers and professionals had a significantly higher scores while manual workers, who were few, showed the highest mean scores although the difference was statistically non-significant. In the education and ‘other’ sectors, routine non-manuals showed the lowest levels of emotional exhaustion when adjusting for age and gender (model 1). In the education sector, there were no manual workers. Adjustment for job demands attenuated the associations in the education sector, while in the health and social care sector the differences between manual workers and other occupational classes widened (model 2). Adjusting for job control widened the differences between occupational groups in the education and ‘other’ sectors (model 3). In health and social care, the differences widened between managers and professionals and the middle occupational classes. In the social and health care sector, managers and professionals had the highest, and routine non-manuals and semi-professionals the lowest levels of emotional exhaustion when adjusting for job strain (model 4). In the education sector, routine non-manuals had the lowest and managers and professionals the highest levels of emotional exhaustion. In the ‘other’ sector, manual workers and routine non-manuals had significantly lower levels of emotional exhaustion than managers and professionals and semi-professionals.

**Table 4.** Emotional exhaustion mean scores with 95% confidence intervals by occupational class according to employment sector adjusted for age, gender and psychosocial working conditions.

Model 1	Health and Social Care	Education	Other
Managers and professionals	2.92, 2.81–3.01	3.01, 2.94–3.08	2.65, 2.55–2.76
Semi-professionals	2.71, 2.66–2.76*	3.03, 2.94–3.08	2.55, 2.43–2.67
Routine non-manuals	2.90, 2.82–2.98	2.89, 2.81–2.97*	2.44, 2.33–2.54*
Manual workers	2.98, 2.55–3.42		2.61, 2.49–2.72
Model 2			
Managers and professionals	2.86, 2.78–2.95	3.02, 2.96–3.08	2.63, 2.54–2.72
Semi-professionals	2.74, 2.70–2.79*	2.94, 2.86–3.02	2.60, 2.49–2.70
Routine non-manuals	2.85, 2.79–2.92	2.94, 2.88–3.01	2.42, 2.33–2.51*
Manual workers	3.12, 2.75–3.50		2.61, 2.51–2.71
Model 3			
Managers and professionals	3.01, 2.92–3.12	3.10, 3.02–3.17	2.72, 2.61–2.83
Semi-professionals	2.71, 2.66–2.76*	3.02, 2.93–3.11	2.56, 2.45–2.68*
Routine non-manuals	2.84, 2.76–2.92*	2.78, 2.69–2.86*	2.42, 2.32–2.52*
Manual workers	2.91, 2.48–3.34		2.54, 2.42–2.65*
Model 4			
Managers and professionals	3.05, 2.96–3.13	3.16, 3.10–3.22	2.77, 2.67–2.86
Semi-professionals	2.74, 2.69–2.79*	2.96, 2.88–3.04*	2.62, 2.52–2.73*
Routine non-manuals	2.74, 2.67–2.81*	2.75, 2.67–2.82*	2.39, 2.30–2.49*
Manual workers	2.88, 2.50–3.26		2.45, 2.35–2.55*

Model 1 Age and gender.

Model 2 Model 1+ job demands.

Model 3 Model 1+ job control.

Model 4 Model 1 + job strain.

*significantly (*p*<0.05) different from the manager and professionals.

## Discussion

The aim of this study was to examine occupational class differences in emotional exhaustion and whether the examined associations vary between employment sectors among municipal employees, and to which extent psychosocial working conditions contribute to the examined associations. We hypothesized that higher occupational class is associated with increased emotional exhaustion and that the employment sector modifies the association and expected that the associations are partly mediated by psychosocial working conditions. In line with our hypotheses, The highest occupational class, i.e. managers and professionals, had the highest scores of emotional exhaustion. Of the sectors, the education sector had the highest scores of emotional exhaustion. The associations between occupational class and emotional exhaustion differed somewhat between the sectors. Adjustment for job demands explained the differences between occupational classes in emotional exhaustion, whereas adjustment for job control and strain widened the differences with some variance across employment sectors.

### Interpretation of the Results

In this cohort of municipal employees, managers and professionals showed more emotional exhaustion than the lower occupational classes, manual workers having the lowest scores. However, the differences between occupational classes were relatively small in magnitude. The few previous studies examining occupational class differences in emotional exhaustion and burnout have shown varying associations. A study conducted among midlife and ageing employees of the City of Helsinki (Helkavaara et al., 2011), showed higher emotional exhaustion among managers and professional as well among those with low job control similar to the present results. In contrast, another Finnish study conducted among the general population showed that among both women and men lower occupational class was associated with higher burnout symptoms, i.e. cynicism and lack of professional efficacy among both women and men, and emotional exhaustion among women (Ahola et al., 2006). In two Swedish studies, women in higher occupational classes have shown lower burnout symptoms (Soares et al., 2007; Norlund et al., 2010). A study conducted in Switzerland showed that higher occupational class was associated with increased burnout and exhaustion while lower occupational class associated with poorer self-rated health and increased sickness absence (Hämmig & Bauer, 2013).

In this study, we also examined the contribution of psychosocial working conditions to the occupational class differences in emotional exhaustion showing higher exhaustion among the managers and professionals in the age and gender adjusted model. If the psychosocial working conditions are advantageous for the lower occupational classes (e.g. low job demands), their adjustment will attenuate the differences, and if they are advantageous to the higher occupational classes (e.g. high job control), their adjustment will widen the differences. Adjustment for job demands explained the differences between occupational classes in emotional exhaustion, whereas adjustment for job control and strain widened the differences. It has been shown also in previous studies that different psychosocial working conditions may have the opposite effects on occupational class differences in mental health outcomes (Hämmig & Bauer, 2013; Qiu et al., 2012), general health outcomes (Rahkonen et al., 2006) as well as physical health across employment sectors (Lahelma et al., 2009). In this study the manual workers had lower job demands than the other occupational classes explaining the differences in emotional exhaustion found between the occupational classes. The managers and professionals had higher job control than the lower classes and thus, adjustment for job control had the opposite effect to adjusting for job demands, widening the differences between occupational classes in emotional exhaustion. Similarly, adjusting for job strain led to widening of occupational class differences as high strain was less common among managers and professionals compared to the other occupational classes. Occupations and occupational classes have different exposures to adverse working conditions with varying health effects (Johnsson et al., 2005). Among higher occupational classes better job control may reduce stress and thereby protect against exhaustion whereas increased job demands may cause stress and predispose to exhaustion. Lower occupational classes, on the other hand, typically have less control over their work, which may also lead to stress and exhaustion while less demanding job protects against exhaustion. Having high strain due to low control and high demands is less common among higher occupational classes protecting against exhaustion. This study examined only part of the complexity around the issue and other working conditions such as work arrangements and job security (Hämmig & Bauer, 2013) as well as physical work characteristics (Helkavaara et al., 2011) are of importance.

The results showed differences in the associations between occupational class and emotional exhaustion by employment sector. Different occupations cannot be directly compared but some indication may be given by analysing occupational class differences by employment sectors. In health and social care, managers and professionals include occupations such as doctors and clinical psychologists. Among semi-professionals, who had the lowest emotional exhaustion, nurse and physical therapist are typical occupations in the health and social care sector. Routine non-manuals include care workers and dental nurses, while manual workers include occupations such as cleaners and instrument technicians. These manual occupations were few in number in these data and therefore the results should be interpreted with caution. In the health and social care sector, sickness absence due to mental disorders is generally more common than in other sectors such as education (Kokkinen et al., 2019; Heinonen et al., 2021), but self-reported emotional exhaustion was lower than in the education sector in this study. In the education sector, emotional exhaustion showed the highest scores in general, but differences between occupational classes were small. However, higher scores of exhaustion among managers and professionals in the educational sector, such as in primary school teachers, is in line with previous studies, e.g. a Norwegian study which showed an increased burnout risk in teachers (Innstrand et al., 2011). Furthermore, similar to our findings, the same study showed increased burnout among doctors and lower burnout among nurses. A study conducted in Belgium showed, however, lower burnout among health and social care than among the other examined sectors (Van den Broeck et al., 2017). The lowest exhaustion within education sector in our study was seen among routine non-manual employees such as childcare workers and classroom assistants. In the ‘other’ sector, emotional exhaustion showed the lowest scores in general and also in this group routine non-manual employees such as firefighters and youth counsellors showed the lowest scores, whereas managers and professionals such as architects and engineers had higher scores.

The majority of the relatively low number of manual workers in these data (n = 268) worked in the ‘other’ sector, as only a few worked in health and social care and none in education, limiting the possibility to draw firm conclusions. The lower emotional exhaustion among manual workers found in the analyses examining all sectors combined can thus be due to differences found across employment sectors, as the few manual workers in health and social care and the ‘other’ sector had higher emotional exhaustion than the middle classes within these sectors. Furthermore, the occupational classes especially in the health and social care and education sectors largely represent the exhaustion among single occupations; in the education sector, for example, the managers and professionals are mainly primary school teachers, and semi-professionals are kindergarten teachers, both having high exhaustion. Exhaustion and the risk of burnout being high among teachers is well established (Innstrand et al., 2011), and widely examined also in Finland (see e.g. Santavirta et al., 2007; Saloviita & Pakarinen, 2021), suggesting that high job strain (Santavirta et al., 2007) is an important factor. A recent study by Saloviita and Pakarinen (2021) showed that additional help for teachers could reduce exhaustion and burnout whilst class size was not an important factor (Saloviita & Pakarinen, 2021).

## Strengths and Limitations

Our study was cross-sectional which limits causal inference and prevents the chronological order of occupational class and psychosocial working conditions and emotional exhaustion to be established. The associations could be bidirectional, although it is more likely that occupational class and psychosocial factors precede emotional exhaustion (Shahidi et al., 2022). We were able to examine occupational class differences among three large employment sectors, i.e. health and social care, education and the ‘other’ sector; however, all participants worked for the same public sector municipal employer. Occupational class differences and psychosocial working conditions might show more variation if the data were representative of the whole workforce e.g. including the private sector. Since our data represent younger municipal employees, generalization of the current findings to private sector employees or to older employees cannot be made. Our advantage is that comparisons of occupational class differences in emotional exhaustion and psychosocial working conditions as explanatory factors could be conducted across the three employment sectors. The strengths of this study also include a large data with both women and men from a variety of occupations. Although the data are female dominated, the gender distribution matches that of the target population (Lallukka et al., 2020), and the municipal sector in Finland.

The response rate to our survey was 51.5%. The non-response analysis showed that the data are broadly representative of the target population with respect to key variables, i.e., sociodemographic and work-related factors and health, although, lower occupational class men were slightly under-represented among the respondents (Lallukka et al., 2020). Thus, it is unlikely that the differences in non-response could substantially bias the associations examined in this study. Nevertheless, it should be acknowledged that the non-response was substantial and needs to be considered when drawing conclusions from the analyses.

The information was based on self-reports, so reporting bias, response styles and personality traits may have affected the results. Some employees who report emotional exhaustion may be prone to report also poor psychosocial working conditions. Such negative affectivity has been found to slightly influence health inequalities and their explanations (Laaksonen et al., 2007). This kind of response style might somewhat overestimate the observed associations, although associations may also be underestimated as some people with health problems may have left employment. Information on occupation as well as on the sector was derived from the employer’s personnel register for those giving consent to record linkage (83%), and from the survey data for the rest, which somewhat avoids reporting bias related to these factors. Nonetheless, working conditions and emotional exhaustion were self-reported. In Finland, the incidence of burnout is difficult to follow from registers, as burnout is not a medical diagnosis. For instance, in occupational health care, depression is often the diagnosis given for those with burnout symptoms (Sauni et al., 2011).

### Implications and Further Research

Burnout is generally more common among women, and this was the finding in our study as well. The present data are female dominated which should be kept in mind, and further studies are warranted to address issues out of the scope of this study. For instance, factors related to family and other factors outside work such as lifestyles may be interesting to study in relation to gender differences. Occupational class differences in emotional exhaustion varied between employment sectors in this study, however, the crude classification of occupational classes may not be comparable between sectors and does not capture qualitative differences between occupational classes. Therefore, a more detailed examination of occupations would be useful in this regard. However, while individuals experience burnout, the results of this study support the notion that reasons leading to burnout are related to the organizations and the work units as well as to the society structures at large. A broader perspective including individual and organizational-level factors on improving wellbeing at work could prove useful and needs further examination (Teoh et al., 2020). In addition, in contemporary societies, the boundaries between work and nonwork may be fading in some occupations and even more so as a result of the Covid-19 pandemic, which forced people in many occupations to work remotely from home (Kerman et al., 2021).

## Conclusions

Examining occupational class differences in emotional exhaustion among public sector employees showed that managers and professionals experience the highest levels of emotional exhaustion. The occupational class differences somewhat differed between the employment sectors, with those working in the education sector having the highest emotional exhaustion scores. A particular attention should be paid to occupations with excess mental demands, and to employees in the education sector, who showed the highest risk of emotional exhaustion.

## References

[bibr1-00332941221106393] Ahola K. Honkonen T. Isometsä E. Kalimo R. Nykyri E. Koskinen S. Aromaa A. Lönnqvist J. (2006). Burnout in the general population. Results from the Finnish health 2000 study. Soc Psychiatry Psychiatr Epidemiol, 41(1), 11–17. 10.1007/s00127-005-0011-5 16341826

[bibr2-00332941221106393] Ahola K. Kivimäki M. Honkonen T. Virtanen M. Koskinen S. Vahtera J. Lönnqvist J. (2008). Occupational burnout and medically certified sickness absence: A population-based study of Finnish employees. J Psychosom Res, 64(2), 185–193. 10.1016/j.jpsychores.2007.06.022 18222132

[bibr3-00332941221106393] Aronsson G. Theorell T. Grape T. Hammarström A. Hogstedt C. Marteinsdottir I. Skoog I. Träskman-Bendz L. Hall C. (2017). A systematic review including meta-analysis of work environment and burnout symptoms. BMC Public Health, 17(1), 264. 10.1186/s12889-017-4153-7 28302088 PMC5356239

[bibr4-00332941221106393] Demerouti E. Le Blanc P. M. Bakker A. B. Schaufeli W. B. Hox J. (2009). Present but sick: A three‐wave study on job demands, presenteeism and burnout. Career Development International, 14(1), 50–68. 10.1108/13620430910933574

[bibr5-00332941221106393] Gluschkoff K. Elovainio M. Kinnunen U. Mullola S. Hintsanen M. Keltikangas-Järvinen L. Hintsa T. (2016). Work stress, poor recovery and burnout in teachers. Occupational Medicine, 66(7), 564–570. 10.1093/occmed/kqw086 27412428

[bibr6-00332941221106393] Hakanen J. J. Schaufeli W. B. (2012). Do burnout and work engagement predict depressive symptoms and life satisfaction? A three-wave seven-year prospective study. J Affect Disord, 141(2–3), 415–424. 10.1016/j.jad.2012.02.043 22445702

[bibr7-00332941221106393] Hämmig O. Bauer G. F. (2013). The social gradient in work and health: A cross-sectional study exploring the relationship between working conditions and health inequalities. BMC Public Health, 13(1), 1170. 10.1186/1471-2458-13-1170 24330543 PMC4028882

[bibr8-00332941221106393] Heinonen N. Lallukka T. Lahti J. Olli P. Nordquist H. Mänty M. Katainen A. Kouvonen A. (2021). Working conditions and long-term sickness absence due to mental disorders: A prospective record linkage cohort study among 19- to 39-year-old female municipal employees. J Occup Environ Med, Publish Ahead of Print. 10.1097/JOM.0000000000002421 PMC8812422 34723911

[bibr9-00332941221106393] Helkavaara M. Saastamoinen P. Lahelma E. (2011). Psychosocial work environment and emotional exhaustion among middle-aged employees. BMC Res Notes, 4, 101. 10.1186/1756-0500-4-101 21463503 PMC3078868

[bibr10-00332941221106393] Hoven H. Siegrist J. (2013). Work characteristics, socioeconomic position and health: A systematic review of mediation and moderation effects in prospective studies. Occup Environ Med. Sep, 70(9), 663–669. 10.1136/oemed-2012-101331 PMC3756612 23739492

[bibr35-00332941221106393] Innstrand S. T. Langballe E. M. Falkum E. Aasland O. G. (2011). Exploring within- and between-gender differences in burnout. 8 different occupational groups. Int Arch Occup Environ Health, 84(7), 813-824. 10.1007/s00420-011-0667-y. 21688002

[bibr11-00332941221106393] Johnson S. Cooper C. Cartwright S. Donald I. Taylor P. Millet C. (2005). The experience of work‐related stress across occupations. Journal of Managerial Psychology, 20(2), 178–187. 10.1108/02683940510579803

[bibr12-00332941221106393] Kalimo R. Toppinen S. (1997). Työuupumus Suomen työikäisellä väestöllä. Helsinki: Työterveyslaitos. [in Finnish].

[bibr13-00332941221106393] Karasek R. Brisson C. Kawakami N. Houtman I. Bongers P. Amick B. (1998). The job content questionnaire (JCQ): An instrument for internationally comparative assessments of psychosocial job characteristics. J Occup Health Psychol, 3(4), 322–355. 10.1037//1076-8998.3.4.322 9805280

[bibr14-00332941221106393] Kerman K. Korunka C. Tement S. (2021). Work and home boundary violations during the COVID-19 pandemic: The role of segmentation preferences and unfinished tasks. Applied Psychology, 1, 23. 10.1111/apps.12335. PMC8444894 34548734

[bibr15-00332941221106393] Kokkinen L. Kouvonen A. Buscariolli A. Koskinen A. Varje P Väänänen A. (2019). Human service work and long-term sickness absence due to mental disorders: A prospective study of gender-specific patterns in 1,466,100 employees. Ann Epidemiol, 31, 57–61. 10.1016/j.annepidem.2018.12.006 30665826

[bibr16-00332941221106393] Kristensen T. S. Borritz M. Villadsen E. Christensen K. B. (2005). The copenhagen burnout inventory: A new tool for the assessment of burnout. Work & Stress, 19(3), 192–207. 10.1080/02678370500297720

[bibr36-00332941221106393] Laaksonen E. Martikainen P. Lahelma E. Lallukka T. Rahkonen O. Head J. Marmot M. (2007). Socioeconomic circumstances and common mental disorders among Finnish and British public sector employees: evidence from the Helsinki Health Study and the Whitehall II Study. Int J Epidemiol, 36(4), 776-86. 10.1093/ije/dym074. 17517811

[bibr17-00332941221106393] Lahelma E. Laaksonen M. Aittomäki A. (2009). Occupational class inequalities in health across employment sectors: The contribution of working conditions. Int Arch Occ Env Hea, 82(2), 185–190. 10.1007/s00420-008-0320-6 18386045

[bibr18-00332941221106393] Lallukka T. Pietiläinen O. Jäppinen S. Laaksonen M. Lahti J. Rahkonen O. (2020). Factors associated with health survey response among young employees: A register-based study using online, mailed and telephone interview data collection methods. BMC Public Health, 20(1), 184. 10.1186/s12889-020-8241-8 32024488 PMC7003443

[bibr19-00332941221106393] Maslach C. Jackson S. E. (1981). The measurement of experienced burnout. Journal of Organizational Behavior, 2(2), 99–113. 10.1002/job.4030020205

[bibr20-00332941221106393] Norlund S. Reuterwall C. Höög J. Lindahl B. Janlert U. Birgander L. S. (2010). Burnout, working conditions and gender--results from the northern Sweden MONICA Study. BMC Public Health, 10, 326. 10.1186/1471-2458-10-326. 20534136 PMC2896942

[bibr21-00332941221106393] Qiu H. Bures R. Shehan C. L. (2012). The inconsistent mediating effects of psychosocial work characteristics on the education-health relationship. Social Science & Medicine, 75(8), 1539–1546. 10.10,16/j.socscimed.2012.06.008 22800919

[bibr22-00332941221106393] Rahkonen O. Laaksonen M. Martikainen P. Roos E. Lahelma E. (2006). Job control, job demands or social class? The impact of working conditions on the relationship between social class and health. Journal of Epidemiology and Community Health, 60(1), 50–54. 10.1136/jech.2005.035758 16361454 PMC2465523

[bibr23-00332941221106393] Saloviita T. Pakarinen E. (2021). Teacher burnout explained: Teacher-student-and organisation-level variables. Teaching and Teacher Education, 97, 103221. 10.1016/j.tate.2020.103221.

[bibr24-00332941221106393] Salvagioni D. A. J. Melanda F. N. Mesas A. E. González A. D. Gabani F. L. Andrade S. M. (2017). Physical, psychological and occupational consequences of job burnout: A systematic review of prospective studies. PLoS One, 12(10), Article e0185781. 10.1371/journal.pone.0185781 28977041 PMC5627926

[bibr25-00332941221106393] Santavirta N. Solovieva S. Theorell T. (2007). The association between job strain and emotional exhaustion in a cohort of 1,028 Finnish teachers. British Journal of Educational Psychology, 77(Pt 1), 213–228. 10.1348/000709905X92045 17411496

[bibr26-00332941221106393] Sauni R. Lagerstedt R. Ahola K. (2011). Miten tyouupumusta hoidetaan tyoterveyshuollossa? Lääkärilehti, 66(14), 1212–1213. [in Finnish].

[bibr27-00332941221106393] Schaufeli W. Enzmann D. (1998). The burnout companion to study and practice: A critical analysis. Lontoo: Taylor & Francis.

[bibr28-00332941221106393] Schaufeli W. B. Desart S. De Witte H. (2020). Burnout Assessment Tool (BAT)—development, validity, and reliability. International Journal of Environmental Research and Public Health, 17(24), 9495. 10.3390/ijerph17249495 33352940 PMC7766078

[bibr29-00332941221106393] Seidler A. Thinschmidt M. Deckert S. Then F. Hegewald J. Nieuwenhuijsen K. Riedel-Heller S. G. (2014). The role of psychosocial working conditions on burnout and its core component emotional exhaustion - a systematic review. J Occup Med Toxicol, 9(1), 10. 10.1186/1745-6673-9-10 24628839 PMC4233644

[bibr30-00332941221106393] Shahidi F. V. Smith P. M. Oudyk J. Gignac M. A. M. (2022). Longitudinal reciprocal relationships between the psychosocial work environment and burnout. Journal of Occupational and Environmental Medicine, 64(3), 226–235. 10.1097/JOM.0000000000002396 35244087

[bibr31-00332941221106393] Soares J. J. Grossi G. Sundin O. (2007). Burnout among women: Associations with demographic/socio-economic, work, life-style and health factors. Arch Womens Ment Health, 10(2), 61–71. 10.1007/s00737-007-0170-3 17357826

[bibr32-00332941221106393] Teoh K. R. H. Hassard J. Cox T. (2020). Individual and organizational psychosocial predictors of hospital doctors’ work-related well-being: A multilevel and moderation perspective. Health Care Manage Rev, 45(2), 162–172. 10.1097/HMR.0000000000000207 29957704

[bibr33-00332941221106393] Toppinen-Tanner S. Kalimo R. Mutanen P. (2002). The process of burnout in white-collar and blue-collar jobs: Eight-year prospective study of exhaustion. Journal of Organizational Behavior, 23(5), 555–570. 10.1002/job.155

[bibr34-00332941221106393] Van den Broeck A. Elst T. V. Baillien E. Sercu M. Schouteden M. De Witte H. Godderis L. (2017). Job demands, job resources, burnout, work engagement, and their relationships: An analysis across sectors. J Occup Environ Med, 59(4), 369–376. 10.1097/JOM.0000000000000964 28157768

